# A method for screening the suppressor genes of siRNA and piRNA pathways using cultured silkworm cells

**DOI:** 10.17912/micropub.biology.000953

**Published:** 2023-09-15

**Authors:** Haruka Sugiyama, Susumu Katsuma

**Affiliations:** 1 Department of Agricultural and Environmental Biology, Graduate School of Agricultural and Life Sciences, The University of Tokyo, Tokyo, Japan

## Abstract

The BmN-4 cell line originates from the ovaries of silkworm, *Bombyx mori* , and possesses endogenous small interfering RNA (siRNA) and PIWI-interacting RNA (piRNA) pathways. BmN-4 cells are latently infected with *Bombyx mori latent virus* (BmLV), an RNA virus whose replication is strictly controlled by both siRNA and piRNA pathways. Knockdown or knockout of the core factors of these two small RNA pathways increases BmLV RNA amount, which in turn inhibits cell growth. Here, we used the known RNAi suppressor CrPV-1A to assess whether the BmN-4 cell line can be used for screening the suppressors of siRNA and piRNA pathways.

**Figure 1. A screening method of the suppressors of small RNA pathways using cultured silkworm cells:**
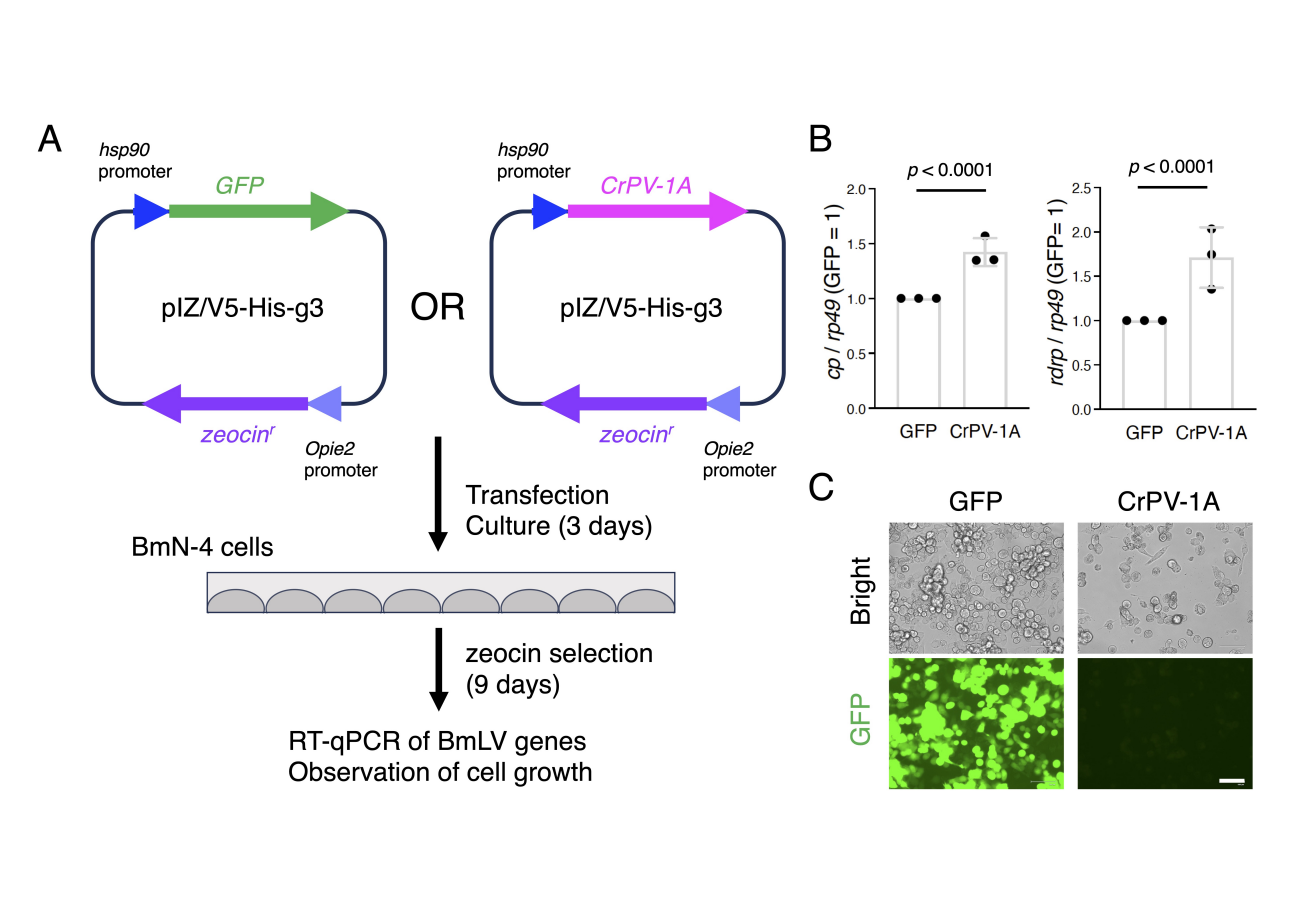
(A) An experimental flow. (B) RT-qPCR results for the BmLV *cp* and *rdrp* genes. BmN-4 cells were transfected with a plasmid expressing GFP- or CrPV-1A, cultured in zeocin-containing medium, and then subjected to RT-qPCR of two BmLV genes. The *cp* and *rdrp* mRNA levels were normalized to that of *B. mori* *rp49* . The data are shown as means ± standard deviation of three independent experiments. *p* -values were calculated via one sample *t* test (two-tailed). (C) Fluorescence microscopy of BmN-4 cells stably expressing GFP or CrPV-1A. The scale bar represents 100 μm.

## Description

Cultured cell lines are an important biological resource for producing recombinant proteins and evaluating gene function. The silkworm *Bombyx mori* cell line BmN-4 is a well-known ovary-derived cell line (Grace, 1967) that has been routinely used for protein production by a baculovirus expression system (Maeda et al. 1985) . In 2009, our group identified PIWI proteins and PIWI-interacting RNAs (piRNAs) that were endogenously expressed in BmN-4 cells. This was the first discovery of a cultured cell line that expressed PIWI/piRNA complexes (Kawaoka et al. 2009) . Many groundbreaking studies involving piRNA factors used this piRNA-producing cell line (Kawaoka et al. 2011; Izumi et al. 2020; Matsumoto et al. 2016) , and it has become a valuable resource for small RNA research (Tsukioka et al. 2006; Kawaoka et al. 2009) .

*Bombyx mori latent virus* (formerly known as *Bombyx mori macula-like virus* , BmMLV) is a positive, single-stranded insect RNA virus that is closely related to plant maculaviruses. BmLV was first discovered in BmN-4 cells, and has been found to infect almost all *B. mori* -derived cultured cell lines (Katsuma et al. 2005) . Surprisingly, BmLV accumulates to extremely high levels (approximately 15% of total mRNA) in BmN-4 cells (Katsuma et al. 2018) . Knockdown or knockout of the core biogenesis genes for either small interfering RNA (siRNA) and piRNA revealed that disruption of these small RNA pathways results in increased BmLV accumulation and inhibition of BmN-4 cell growth (Katsuma et al. 2018; Katsuma et al. 2021) . These findings show that the siRNA and piRNA pathways function cooperatively to silence BmLV RNA and that both pathways are required for the normal growth of BmLV-infected silkworm cells.

In this study, we tested whether BmN-4 cells can be used to assess the suppressor activity of foreign genes by measuring the expression levels of BmLV genes and observing the degree of inhibition of cell growth. We selected the cricket paralysis virus 1A protein (CrPV-1A) gene as the suppressor gene. CrPV-1A has been found to inhibit Ago2-dependent RNAi via blocking the initial target searching by Ago2-RISC (Watanabe et al. 2017; Nayak et al. 2018) .

First, we cloned *CrPV-1A* into the vector, pIZ/His-V5-g3, so that the cloned gene would be expressed under the control of the *B. mori hsp90* promoter (Hirota et al., 2021) . Next, pIZ/His-V5-g3-CrPV-1A or pIZ/His-V5-g3-GFP (i.e., control vector) was transfected into BmN-4 cells and treated with zeocin from 3 days after transfection ( Fig. 1A ). Selection was conducted for 9 days, after which cells were photographed and then collected for RNA isolation ( Fig. 1A ). Reverse transcription-quantitative polymerase chain reaction (RT-qPCR) experiments revealed that CrPV-1A expression increased the mRNA levels of two BmLV genes, *coat protein* ( *cp* ) and *RNA-dependent RNA polymerase* ( *rdrp* ) ( Fig. 1B ), suggesting that CrPV-1A may block one or both of the siRNA and piRNA pathways. In addition, CrPV-1A expression also strongly inhibited the cell growth of BmN-4 cells ( Fig. 1C ). Taken together, these results demonstrate that BmN-4 cells can be used for a simple screening system to identify novel putative suppressors of the siRNA and/or piRNA pathways.

## Methods

**Cell line**

BmN-4 cells (provided by Chisa Yasunaga-Aoki, Kyushu University, and maintained in our laboratory) (Maeda et al. 1985) were cultured at 27°C in IPL-41 medium (Applichem) supplemented with 10% fetal bovine serum.

**Plasmid construction**

A CrPV-1A fragment was amplified from pCold II-CrPV-1A (provided by Yukihide Tomari) (Watanabe et al. 2017) . This fragment was then cloned into the vector pIZ/V5-His-g3 (Hirota et al., 2021) using the In-Fusion HD Cloning Kit (Clontech). pIZ/V5-His-g3-GFP (Hirota et al., 2021) was used as a control.

**Transfection and generation of stably transfected BmN-4 cells**

BmN-4 cells (2 × 10 ^5^ cells per 35 mm dish) were transfected with 1 µg of pIZ/V5-His-g3-GFP or pIZ/V5-His-g3-GFP-CrPV-1A using FuGENE HD (Promega). Three days after transfection, zeocin (InvivoGen, final concentration of 500 µg/mL) was added to the medium (Kawaoka et al. 2009) . Nine days after drug selection, cells were observed using a FLoid ^TM^ cell imaging station (Life Technologies). Thereafter they were collected for RNA extraction.

**RT-qPCR**

Total RNA was isolated using TRI Reagent® (Sigma-Aldrich) and then subjected to reverse transcription with avian myeloblastosis virus reverse transcriptase and an oligo-dT primer (TaKaRa). RT-qPCR was performed using a KAPA SYBR FAST qPCR kit (Kapa Biosystems) and the specific primers. The expression values were calculated using the 2 ^−ΔΔCt^ method.

## Reagents

PCR primers for CrPV-1A

CrPV1A-f: TACCGAGCTCGGATCatgtcttttcaacaaacaaacaacaacgc

CrPV1A-r: GCCACTGTGCTGGATctagaaggctctgcattcatcattac

qPCR primers for BmLV *cp*

coat-2F: TCCTCTCGCATTACTATTGG

coat-2R: ATGGAGCCTCTGATGACAAC

qPCR primers for BmLV *rdrp*

rdrp-2F: TCTCTCATGAAATCAGCACC

rdrp-2R: TCACGATATGGTTTGAGATG

qPCR primers for *B. mori rp49*

rp49-F: CCCAACATTGGTTACGGTTC

rp49-R: GCTCTTTCCACGATCAGCTT
